# Diverse Onchocercidae from Malaysian cats and Indonesian macaques: Morphological and molecular analysis of individual microfilariae using mitochondrial genomes, 28S rRNA, and *Wolbachia* endosymbiont sequences

**DOI:** 10.1371/journal.pntd.0014015

**Published:** 2026-07-23

**Authors:** Irina Diekmann, Young-Jun Choi, Taniawati Supali, Rahmat Alfian, Yossi Destani, Elisa Iskandar, Noviani Sugianto, Mohd Hatta Abdul Mutalip, Nor Azlina Abdul Aziz, Khairiah Ibrahim, Kerstin Fischer, Makedonka Mitreva, Peter U. Fischer

**Affiliations:** 1 Infectious Diseases Division, Department of Medicine, Washington University School of Medicine, St. Louis, Missouri, United States of America; 2 Department of Parasitology, Faculty of Medicine, Universitas Indonesia, Jakarta, Indonesia; 3 Institute for Public Health, National Institute of Health, Setia Alam, Selangor, Malaysia; 4 Department of Veterinary, Pathology and Microbiology, Faculty of Veterinary Medicine, Universiti Putra Malaysia, Selangor, Malaysia; 5 McDonnell Genome Institute, Washington University School of Medicine, St. Louis, Missouri, United States of America; NIAID: National Institute of Allergy and Infectious Diseases, UNITED STATES OF AMERICA

## Abstract

During an investigation of animals as reservoirs for the filarial parasite *Brugia malayi*, three molecularly undescribed filarial species were co-detected. Individual microfilariae (Mf) were isolated and analyzed from blood samples of crab-eating macaques (*Macaca fascicularis*) from Belitung, Indonesia, and from pet dogs and cats in Sabah, Malaysia. Among 163 macaques, 33 (20.2%) were positive for large Mf (mean length 498.9 µm) similar to *Dirofilaria* (‘Belitung I’). One macaque was infected with small Mf (mean length 150.4 µm) (‘Belitung II’), with a high density of 17,150 Mf/mL. In two cats co-infected with *B. malayi*, Mf of a *Dirofilaria* species (‘Sabah’) with an average length of 299.1 µm were detected. Morphometric analysis of Mf showed distinct differences between these three species and other Mf described in the area. Whole genome amplification and genome sequencing of 24 individual Mf enabled phylogenetic analysis of mitochondrial genomes, and analysis of specific mitochondrial and nuclear barcode regions. The three Mf groups formed distinct clusters and could not be identified by comparison with available reference sequences. Cluster ‘Belitung I’ from macaques formed a sister group to other characterized *Dirofilaria*. Cluster ‘Belitung II’ included bird filariae and primate filariae of the genus *Mansonella* as close relatives. The cluster ‘Sabah’ formed a monophyletic group with the zoonotic species *Dirofilaria asiatica* and *Dirofilaria* sp. ‘Thailand’. DNA of *Wolbachia* endobacteria was detected in Mf of ‘Belitung I’ and ‘Sabah’, but not in ‘Belitung II’. These findings highlight the limited understanding of filarial diversity in macaques and cats in Asia and underscore the need for a more comprehensive approach that combines morphological and molecular data to identify and assess the pathogenicity and zoonotic potential of these parasites.

## Author summary

Filarial worms are parasitic nematodes that infect humans and animals and are often transmitted by the same vector mosquito. We identified three molecularly undescribed filarial species while investigating animals as reservoirs for the agent of lymphatic filariasis, *Brugia malayi* on Belitung Island, Indonesia, and in Sabah, Malaysia. Blood samples were collected from Indonesian macaques and Malaysian pet cats. Out of 163 macaques, 20.2% tested positive for exceptionally large microfilariae (Mf) of an unclassified Onchocercidae species (‘Belitung I’). Another filarial species (‘Belitung II’) with very small Mf, but with a remarkably high density of 17,150 Mf/mL was detected in one macaque. Two cats harbored medium sized Mf of a *Dirofilaria* species (Sabah’). Genetic analysis revealed unique phylogenetic clusters that did not match any reference sequence. *Dirofilaria* sp. ‘Sabah’ was closely related to the zoonotic *D. asiatica* complex, whereas ‘Belitung I’ clustered as a sister group to *Dirofilaria*. ‘Belitung II’ Mf clustered next to but not within the *Mansonella spp.* cluster. DNA of *Wolbachia* endobacteria was only detected in Mf of ‘Belitung I’ and ‘Sabah’. These findings highlight the limited understanding of filarial diversity in animals and underscore the need for a comprehensive approach that combines morphological and molecular data to identify and assess the pathogenicity and zoonotic potential of these parasites.

## Introduction

Filarial parasite species are globally distributed among mammalian, reptile, and avian hosts and are classified primarily under the superfamily Filarioidea, which is subdivided into Filariidae, and Onchocercidae [1,2]. Despite their wide host range and diverse tropisms, many species remain poorly characterized. Morphological and molecular species description of members of this superfamily are often missing, and/or available morphological and molecular data cannot be reliably linked.

The limitations of relying solely on molecular data are illustrated by *Dirofilaria* sp. ‘hongkongensis’, which was first detected in a human in 2012 [3]. Although this species has been repeatedly reported in humans and animals in Bhutan, India [4–7], Sri Lanka [8], and Hong Kong [3,9], its taxonomic placement remained unresolved for more than a decade. The detailed morphological descriptions of adult worms and Mf, combined with genomic data, allowed the species *Dirofilaria* sp. ‘hongkongensis’ to be formally named and described as *Dirofilaria asiatica sp.* nov. [10]. *Dirofilaria asiatica* sp. nov., and *D.*sp. ‘Thailand II’ form a closely related monophyletic group [11].

An accurate parasitic nematode and complete species description requires adult nematodes, particularly males, which are often accessible only through host necropsy. Although collecting and retrieving adult nematodes during surgery is feasible in domestic animals, obtaining comparable samples from wild animals, such as primates, is often impossible due to ethical and regulatory concerns. As a result, many filarial species circulating in wildlife remain insufficiently described, with either lacking links between morphological and molecular data or missing both. This gap is concerning because several filarial species are of significant public health relevance. *Brugia malayi*, for example, causes lymphatic filariasis and remains a priority in World Health Organization elimination programs. Other zoonotic species, such as *D. asiatica*, further underscore the importance of a One Health approach that recognizes the interconnectedness of human, animal, and environmental health in understanding and managing infectious diseases. Yet, despite their importance, filarial parasites have not been extensively studied within a One Health framework focused on human and animal health.

We recently demonstrated that non-human primates act as important reservoir hosts for brugian filariasis. On Belitung Island, Indonesia, long-tailed macaques (*Macaca fascicularis*) were identified as the main reservoir of *B. malayi* in an area endemic for human lymphatic filariasis [12]. These macaques are highly adaptable and synanthropic, in contrast to other primate species on the island that are largely nocturnal and avoid human contact [13]. Because macaques may harbor filarial species pathogenic to humans and mosquito vectors may bite both human and non-human primates, close interaction between hosts may facilitate transmission within species and between species [14,15].

The objective of the present study was to provide molecular and morphometric data of Mf found in hosts of *B. malayi* in Southeast Asia to improve differential diagnosis. We examined non-human primates and domestic cats and dogs that live in close proximity to humans and are likely to be bitten by the same mosquito vectors. Whole mitochondrial genomes, together with mitochondrial and nuclear barcode regions, were analyzed to clarify phylogenetic relationships. These data expand existing molecular reference databases and enhance the ability to identify filarial species with zoonotic potential.

## Materials and methods

### Ethics approval and consent

The trapping and blood collection of animals were approved by the Ministries of Health and the Environment and Forestry of Indonesia (protocol #22-040365). The study received ethical approval from the ethical committee of Universitas Indonesia (no 515/UN2.F1/ETIK/PPM.00.02/2022) and was performed by veterinarians or, under their supervision, by veterinary technicians. Blood sample collection in Malaysia was approved by Animal Care and Use Committee (ACUC/KKM/02(01/2024)). Blood collection was performed by veterinarians.

### Study area and sample collection

Animal blood samples were collected as part of a study investigating the role of animal reservoirs for *B. malayi* in Indonesia and Malaysia. Belitung Island, Indonesia, is administratively divided into two districts, Belitung and Belitung Timur. Blood samples were obtained in five areas in the Belitung district (Selat Nasik and Petaling on Mendanau island, and Kembiri, Lassar, and Kacang Butor on the main island Belitung). The study area has been described in detail previously [16]. Macaques were trapped, and samples were collected at different time points (between 07:30–10:30 am or between 3:00–7:30 pm). The sample collection procedures have been described in detail previously [12]. In eastern Malaysia (Sabah, Borneo), pet cats and dogs were examined for Mf in an area with persistent *B. malayi* infection despite mass drug administration in the human population. Five morphologically *B. malayi* positive blood samples were sent to Washington University in St. Louis for further molecular diagnostic confirmation. Samples from two cats also contained Mf of another filarial species. Data on sex, village, sub-village, GPS coordinates, and habitat (forest near residence/ tourist attraction or palm oil plantation) were collected (S1 Table). None of the animals showed clinical signs of filarial infection.

### Morphological description

**Fig 1:**
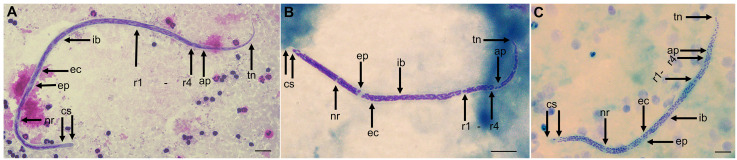
Morphological features of Giemsa-stained microfilaria. **(A)** Onchocercidae gen. sp. ‘Belitung I’. **(B)** Onchocercidae gen. sp. ‘Belitung II*’*. **(C)** *Dirofilaria* sp. ‘Sabah’. Abbreviation: cs-cephalic space; nr-nerve ring; ec-excretory cell; ep-excretory pore; ib-inner body or “Innenkörper”; ap-anal pore; r 1-4-rectal cells; tn-terminal nucleus, scale bar indicates 10 µm.

In Indonesia, two experienced microscopists examined the slides to identify and count Mf. A three-line thick blood smear (60 µl on the slide) was prepared following previously described procedures [17]. The slides were air-dried for 2 days at ambient temperature and then stained with 3% Giemsa solution (Sigma-Aldrich, Germany). Species identification was based on morphological features such as the presence of a sheath, the staining characteristics of the sheath, measurement of total body length and width, cephalic space (length, width and their ratio), the location of the nerve ring (NR), excretory pore (EP), excretory cell (EC), anal pore (AP), genital pore (GP), the “Innenkörper“, and the number and position of terminal nuclei (TN) in the tail structure of the microfilariae [18]. For morphometric comparison, measurements were taken from 15 individual Mf of an unclassified Onchocercidae gen. sp. ‘Belitung I’ from five macaques, 15 Mf of an unclassified Onchocercidae gen. sp. ‘Belitung II’ from a single macaque on Belitung Island, Indonesia, and 13 Mf of the unclassified *Dirofilaria* sp. ‘Sabah’ from one cat in Malaysia. Measurements included the length of the cephalic space clear of nuclei, the distance from the head to the nerve ring, the distance from the head to the excretory pore, the distance from the head to the anal pore, the distance from the head to the tail (terminal nuclei), the width at the level of the nerve ring, the total body length, and the ratio of mid-body width to the length of the nuclei-free cephalic space (Fig 1). All measurements and photographs were taken using an OLYMPUS BX40F4 microscope (Olympus Optical Co. Ltd., Japan) at 1000x magnification with cellSens Standard 1.18 software (Olympus Corporation of the Americas, USA).

### DNA extraction and sequencing

DNA was extracted from 50 µL of each blood sample, and the presence of pan-filarial DNA, specifically *B. malayi*, *B. pahangi,* and *D. immitis*, was assessed by qPCR as described in a previous study [12]. For selected samples that were morphologically microfilariae positive (S1 and S2 Tables), whole-genome amplification and sequencing were performed on individual Mf according to a previously described protocol [19]. Briefly, for DNA isolation from a single mf, we used a modified CGP DNA isolation protocol described by Choi et al. 2024 [19,20]. To confirm a successful DNA isolation, a qPCR assay was performed to amplify a 28S ribosomal RNA pan-filarial fragment. qPCR conditions and primer pairs were described in previous studies [11,20]. Positive samples were subjected to whole genome amplification with random primers using the Ready-To-Go GenomiPhi V3 DNA Amplification Kit (Cytiva, Marlborough, MA) according to the manufacturer’s recommendations. After whole-genome amplification, the sample was diluted 1:10, and the presence of parasite DNA was confirmed by qPCR. DNA concentration was measured by Qubit 4.0 (Thermo Fisher Scientific, Waltham, MA, USA) with the dsDNA BR Assay Kit (Thermo Fisher Scientific). A Kapa Hyper PCR-free library was generated from the amplified DNA and sequenced on Illumina’s NovaSeq platform (San Diego CA, USA, 2 × 150 bp paired-end reads) to ~10 Gb per sample (S2 Table). Short-read sequencing data from whole-genome amplification were not suitable for de novo nuclear genome assembly, and the study focused on assembly and analysis of the mitochondrial genome.

### Mitochondrial genome assembly and phylogenetic analyses

Sequencing reads were adapter- and quality-trimmed using Trimmomatic v0.39 [21]. GetOrganelle v1.7.5 [22] (-R 10 -k 21,45,65,85,105 -F animal_mt) was used to assemble *de novo* mitochondrial DNA from paired-end data. Assembly graphs were assessed using Bandage, and read coverage across the assemblies was evaluated (S1 Fig) [23]. Mitochondrial genomes of *B. malayi* (AF538716) and *D. immitis* (AJ537512) were included in the seed database. MITOS2 v2.1.9 [24] was used to determine the gene complement and the order of the protein-coding genes. The predicted gene models were manually reviewed and curated. The fully circularized assembled genomes and whole mitochondrial genome sequences of *Onchocercidae* retrieved from GenBank were linearized to a common start position using rotate v1.0 [25]. Multiple sequence alignment of the DNA sequences was performed using MAFFT v7.505 [26] (--maxiterate 1000 --globalpair), followed by alignment trimming using trimAl v1.4 [27] (-automated1). A maximum likelihood phylogenetic tree was constructed using IQ-TREE2 v2.2.0 [28] (-B 10000 -bnni -alrt 10000 --robust-phy 0.98 -wsl) with *Spirocerca lupi* (KC305876) and *Tetrameres grusi* (MW648425) as outgroup taxa (-o) and 10,000 ultrafast bootstrap replicates [29]. The best-fit substitution model (GTR + F + I+I + R4) was selected using ModelFinder within IQ-TREE2 [30]. The multiple sequence alignments in PHYLIP format used for phylogenetic tree construction and the corresponding Newick tree files for all phylogenetic analyses are provided in S1 Dataset. The tree was visualized using FigTree (version 1.4.4) [31]. Graphical maps of mitochondrial genomes were generated using OrganellarGenomeDRAW (OGDRAW) v1.3.1 [32].

### Cytochrome *c* oxidase I (COI) and 28S ribosomal RNA gene analyses

To improve taxonomic sampling, given that complete mitochondrial genomes are available for fewer species than reference sequences for commonly used barcoding regions, the cytochrome *c* oxidase I (COI) region from the whole mitochondrial genome sequences of each Mf sample was used to search the NCBI database using BLASTn [33] and to retrieve reference sequences from the family *Onchocercidae* (NCBI:txid6296) (GenBank accessed on 5 November 2025) (S3 Table). *Tetrameres grusi* (MW648425) and *Spirocerca lupi* (KC305876) were included as outgroup. COI sequences were aligned using MAFFT v7.505 [26] and examined for correct codon alignment and in-frame translation using SeqKit translate [34]. Sequences shorter than 495 base pairs that resulted in incomplete coverage of the region were excluded. A maximum likelihood phylogenetic tree was constructed using IQ-TREE2 v2.2.0 [28] (-B 10000 -bnni -alrt 10000 --robust-phy 0.98 -wsl -st CODON5) with a constrained tree search (-g). A whole mitochondrial genome tree was built as described above, and all nodes with bootstrap support below 99% were converted to multifurcating nodes using ggtree [35]. This tree was used as the constraint tree for the COI analysis. The best-fit substitution model (GY + F + R5) was selected using ModelFinder within IQ-TREE2 [30]. The tree was visualized using FigTree (version 1.4.4) [31].

The nuclear-encoded 28S rRNA gene was analyzed using a similar approach. Sequencing data from each Mf sample was assembled individually using SPAdes v4.2.0 [36]. The 28S rRNA region in each assembly was identified using minimap2 [37] with the 28S rRNA sequence from *Eufilaria acrocephalusi* (MT802308) as the query. The coordinates of the aligned region were converted to BED format and then to FASTA format using bedtools v2.31.0 [38]. Comparator sequences from *Onchocercidae* were retrieved from GenBank (S3 Table), aligned using MAFFT v7.505 [26] (--maxiterate 1000 --localpair), and trimmed using trimAl v1.4 [27] (-nogap). A maximum likelihood phylogenetic tree was generated using IQ-TREE2 v2.2.0 [28], as described above, with *Spirocerca lupi* (OZ222510) as the outgroup and without applying a constraint tree. The best-fit substitution model (TVM + F + I+I + R2) was selected using ModelFinder within IQ-TREE2 [30]. The tree was visualized using FigTree (version 1.4.4) [31].

### Analysis of *Wolbachia* endosymbionts

The Chan Zuckerberg ID metagenomic pipeline was used to identify putative *Wolbachia*-derived reads and estimate their relative abundance [39]. Raw sequencing data were submitted to the cloud-based pipeline, which characterized microbial composition by assigning sequencing reads to taxonomic categories. Taxon abundance was reported as reads per million (rPM), defined as the number of reads aligned to a given taxon in the NCBI nucleotide (NT) database per million reads sequenced. This metric normalizes read counts across samples and enables comparisons of the relative abundance of taxa among samples. Multilocus sequencing typing (MLST) gene sequences were used to classify *Wolbachia* into supergroups. *Wolbachia pipientis* MLST sequences for coxA, fbpA, and gatB [40] were retrieved from PubMLST [41]. These sequences were used as queries in Exonerate [42] (--model protein2genome --bestn 1 --showtargetgff --showvulgar no --showalignment yes) to identify the corresponding genes in contigs assembled from individual microfilaria samples using SPAdes v4.2.0 [36]. Coding sequences (CDSs) were extracted from each hit using GffRead (-x) [43]. Multiple sequence alignments were generated using MAFFT v7.505 [26] (--maxiterate 1000 --localpair), trimmed using trimAl v1.4 [27] (-automated1), and concatenated using catfasta2phyml (https://github.com/nylander/catfasta2phy). Phylogenetic analysis was performed using IQ-TREE2 v2.2.0 [28], as described above, with wTex used as the outgroup [44]. The best-fit substitution model (TPM2u+F + I+I + R3) was selected using ModelFinder within IQ-TREE2 [30]. The tree was visualized using FigTree (version 1.4.4) [31].

## Results

### Microscopy-based species identification, morphometric description, and density of microfilariae

In total, 33 of 163 (20.2%) blood samples from macaques contained Mf of an unclassified Onchocercidae gen. sp. (‘Belitung I’) (S1 Table). Among these, nine macaques (5.5%) were co-infected with *B. malayi* (S1 Table). The geometric mean Mf density for Onchocercidae gen. sp. ‘Belitung I’ in macaques was 34 Mf/mL (range 17–484 Mf/mL) (S1 Table). In addition, unclassified Onchocercidae gen. sp. Mf ‘Belitung II’ collected from a male macaque in the forest habitat near a residence in the village of Selat Nasik could not be clearly identified by morphology. This macaque from Selat Nasik showed an exceptionally high Mf density of 17,150 Mf/mL. The arithmetic mean length of the Giemsa-stained Mf of ‘Belitung II’ was 150.4 ± 9.14 µm, whereas the unclassified Onchocercidae gen. sp. ‘Belitung I’ was more than three times larger, with a head-to-tail length of 498.9 ± 13.15 µm. Out of the five samples obtained from Malaysia (from three cats and two dogs), two cats harbored *Dirofilaria* sp. ‘Sabah’ Mf. The head-to-tail length was 299.1 ± 6.52 µm (Table 1). The Mf density of this species was not determined. No sheaths and terminal nuclei were detected in any of the analyzed Mf.

**Table 1:** Morphometry of Giemsa-stained microfilariae in µm. Measurements are presented as range and arithmetic mean ± SD.

	Onchocercidae gen. sp. ‘Belitung I’	Onchocercidae gen. sp. ‘Belitung II’	*Dirofilaria* sp. ‘Sabah’
Host	macaques	macaque	cat
No. of Mf	15	15	13
nuclei in head region	–	–	1-3
length nuclei free cephalic space	13.84 -17.43 | 15.04 ± 0.9	1.61 - 4.4 | 2.7 ± 0.6	9.3 -11 | 10.2 ± 0.54
length head to nerve ring	84.05 - 95.23 | 88.51 ± 2.91	25.57 - 31.5 | 29.56 ± 1.8	64.3- 71.54 | 67.56 ± 2.25
length head to excretory pore	129.4 - 172.5 |140.9 ± 9.89	38.97 - 48.46 | 44.87 ± 2.73	95.2 - 114.8 | 102.1 ± 6.61
length head to anal pore	354.2 - 406.1 | 380.7 ± 14.51	110.1-132.8 | 122.3 ± 7.06	201.5 - 232.2 | 217.9 ± 9.314
length head to tail	480.3 - 523.3 | 498.9 ± 13.15	134.6 - 165.6 | 150.4 ± 9.14	289.8 - 313.4 | 299.1 ± 6.524
midbody width	4.33 - 7.1 | 5.8 ± 0.9	3.16 - 4.91 | 4.028 ± 0.52	5.27 - 8.4 | 6.69 ± 0.81
Ratio-width-nuclei	2.06 - 3.15:1 | 2.65 ± 0.49:1	0.47 - 1.30:1 | 0.68 ± 0.22:1	0.49 - 0.81:1 | 0.66 ± 0.1

### Diagnostic qPCR results

The qPCR results for filarial species originating from macaques have been previously published [12]. None of the macaques tested positive for *B. pahangi* or *D. immitis*. For the Malaysian sample, all samples were pan-filarial positive. Three cat samples were positive for *B. malayi* and *B. pahangi*, including both cats that contained *Dirofilaria* sp. ‘Sabah’. None of the cat samples were positive for *D. immitis*. Only one dog tested positive for *D. immitis* (S1 Table).

### Phylogenetic analyses based on whole mitochondrial genome

Mitochondrial genomes were *de novo* assembled for 24 Mf sample (S2 Table), yielding complete circularized genomes within the expected size range (13.6-13.8 kb) and displaying the conserved set of 12 protein-coding genes and the characteristic gene order of filarial nematodes [45] (S2 Fig). The mitochondrial genome sequences are publicly available in GenBank under the following accession numbers: Onchocercidae sp. ‘Belitung I’ (PX995888 - PX995902), Onchocercidae sp. ‘Belitung II’ (PX995903 - PX995905), and *Dirofilaria* sp. ‘Sabah’ (PX995906 - PX995911) (S2 Table).

To determine the taxonomic placement of the unclassified filarial species, these genomes were compared with mitochondrial sequences of other Onchocercidae taxa available in GenBank (S3 Table). Pairwise nucleotide identity analyses showed that Onchocercidae sp. Belitung I was most similar to *Dirofilaria repens* (KX265048; 88.0% identity; 98% query coverage). Onchocercidae sp. ‘Belitung II’ showed the highest similarity also to *D. repens* (KX265048; 83.3% identity; 98% query coverage), whereas *Dirofilaria* sp. ‘Sabah’ most closely matched *D. asiatica* (PQ131197; 96.9% identity; 100% query coverage).

**Fig 2:**
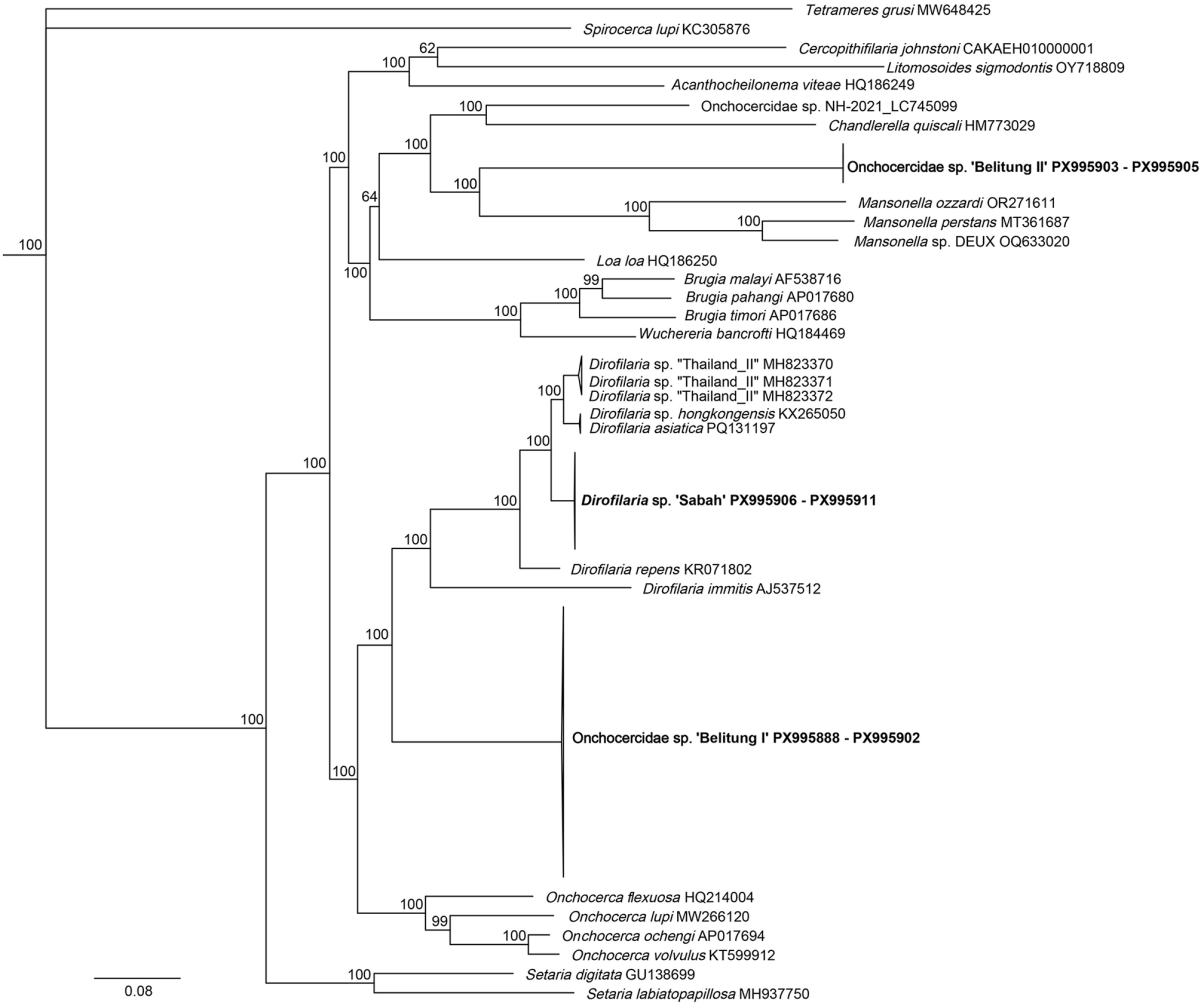
Whole mitochondrial genome maximum likelihood phylogenetic tree. The scale bar represents 0.8 substitutions per site, and node support was estimated by 10,000 ultrafast bootstrap replicates.

A maximum likelihood phylogenetic tree including 23 additional Onchocercidae taxa revealed that Onchocercidae sp. ‘Belitung I’ formed a well-supported clade outside the *Dirofilaria* genus and appeared as its sister group, suggesting that it may represent a distinct lineage (Fig 2). In contrast, *Dirofilaria* sp. ‘Sabah’ clustered within the *Dirofilaria* genus and grouped monophyletically with *D. asiatica* and *Dirofilaria* sp. ‘Thailand II’ [10,11]. Onchocercidae sp. ‘Belitung II’ formed a separate clade that was the sister group to the genus *Mansonella.*

### Phylogenetic analyses based on cytochrome *c* oxidase I (COI) and 28S ribosomal RNA gene

To improve taxonomic resolution, the COI region from each mitochondrial genome was queried against the NCBI database to retrieve reference sequences from the family Onchocercidae (NCBI:txid6296). The COI sequences of Onchocercidae sp. ‘Belitung I’ showed the highest similarity to an unclassified *D. repens*-like species from Georgia, USA (PQ191455; 94.9% identity; 100% query coverage). The closest match to Onchocercidae sp. ‘Belitung II’ COI sequences were *Eufilaria sylviae* (MT800771; 89.7% identity; 100% query coverage), identified from a garden warbler (*Sylvia borin*) in Lithuania. The COI sequences of *Dirofilaria* sp. ‘Sabah’ were most similar to *D. asiatica* (PV523835; 97.4% identity; 100% query coverage), obtained from a dog in Sri Lanka.

Sequence similarity searches using the nuclear-encoded 28S rRNA gene indicated that Onchocercidae sp. ‘Belitung I’ was most similar to *Dirofilaria ursi* (PV389592; 95.9% identity; 100% query coverage), obtained from a Japanese black bear in Japan. The 28S rRNA gene sequences of Onchocercidae sp. ‘Belitung II’ most closely matched *Mansonella perstans* (MN432520; 90.3% identity; 100% query coverage), obtained from a human in Brazil. *Dirofilaria* sp. ‘Sabah’ showed the highest similarity to *D. repens* (KP760376; 98.7% identity; 100% query coverage), obtained from a dog in Italy. The 28S rRNA sequences generated in this study are available under the following GenBank accession numbers: Onchocercidae sp. ‘Belitung I’ (PZ099891 - PZ099893), Onchocercidae sp. ‘Belitung II’ (PZ099888 - PZ099890), and *Dirofilaria* sp. ‘Sabah’ (PZ099894 - PZ099896) (S2 Table).

**Fig 3:**
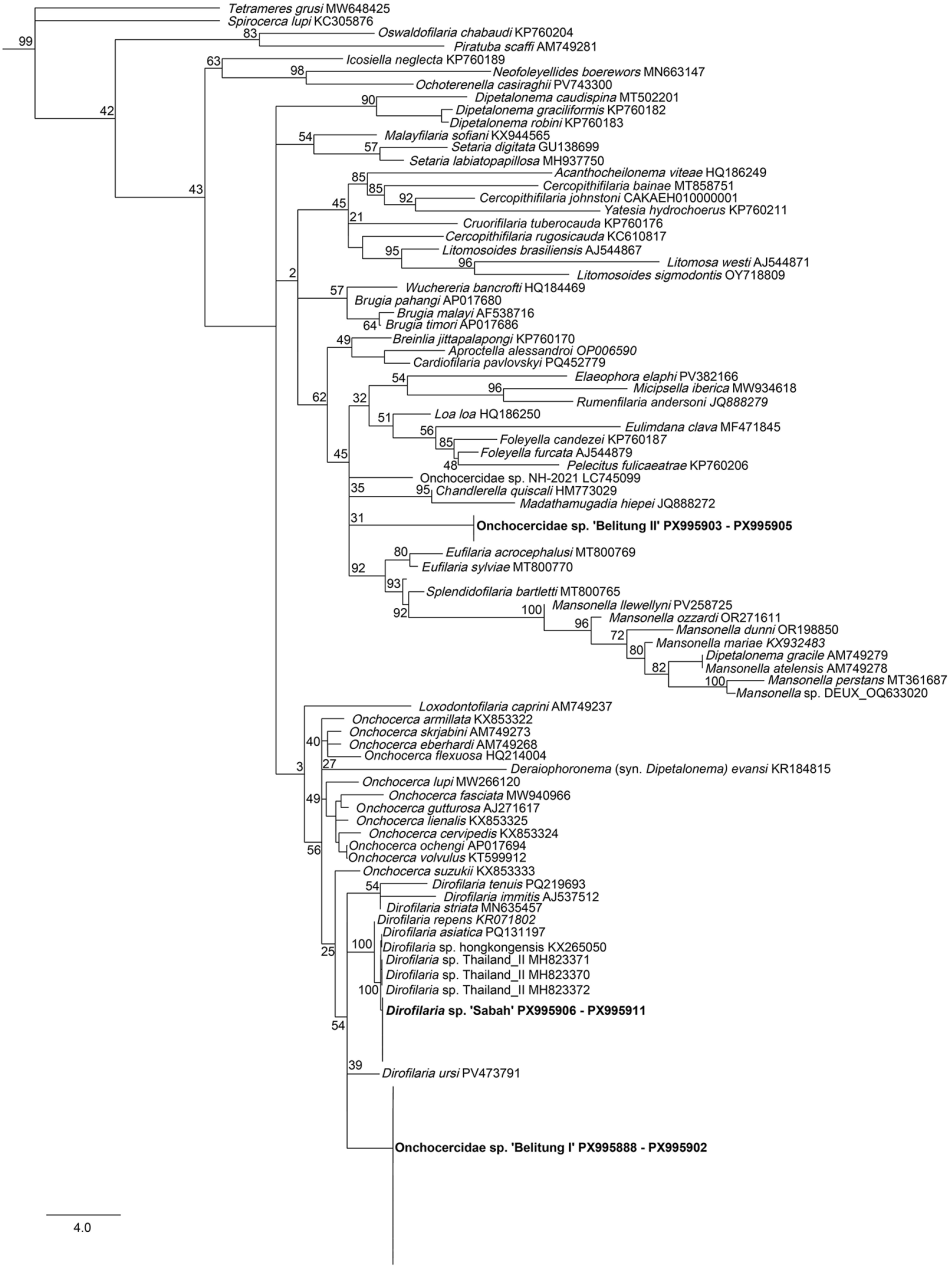
Cytochrome *c* oxidase I maximum likelihood phylogenetic tree. The scale bar represents 4.0 substitutions per site, and node support was estimated by 10,000 ultrafast bootstrap replicates.

**Fig 4:**
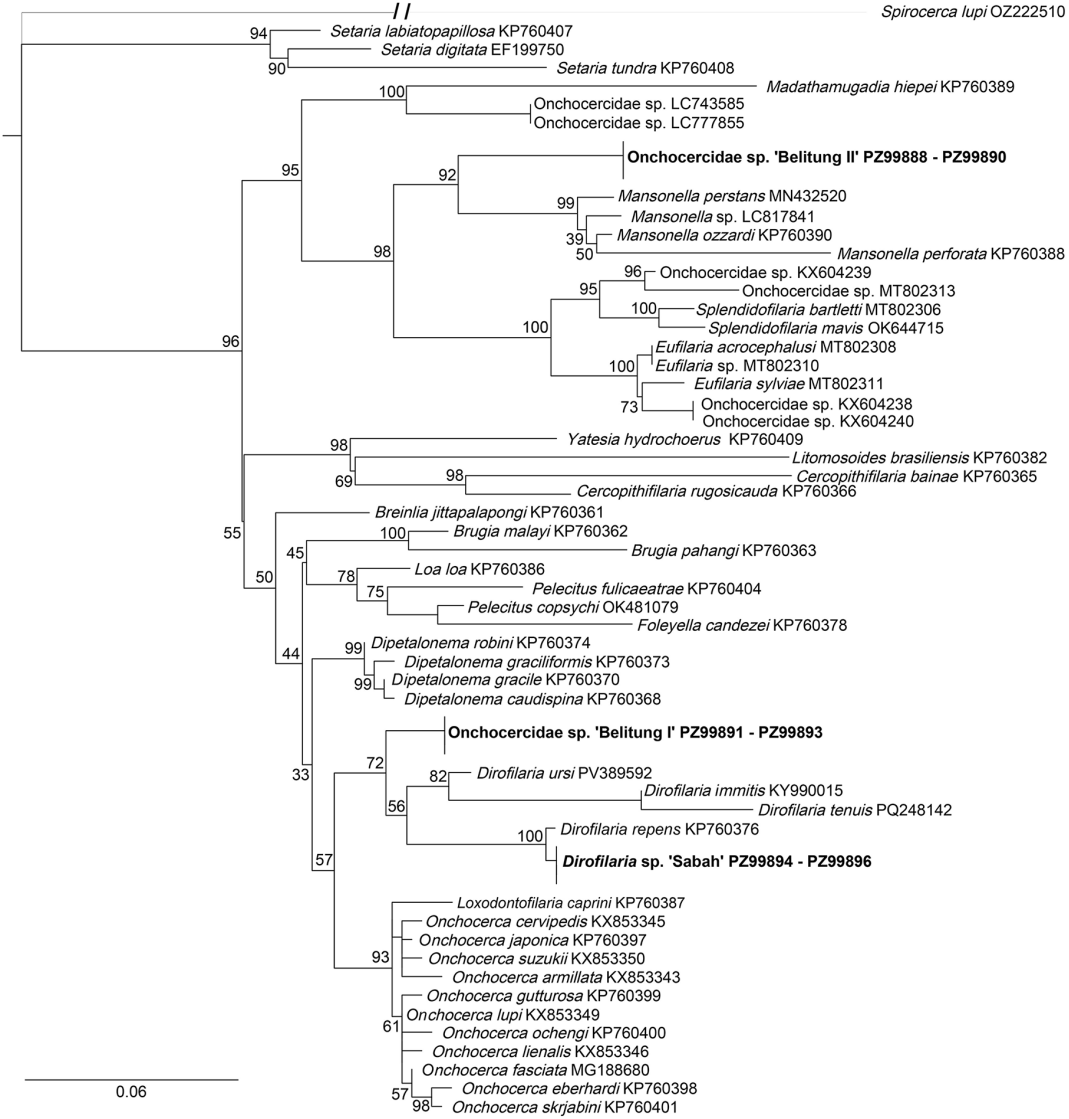
28S ribosomal RNA maximum likelihood phylogenetic tree. The scale bar represents 0.06 substitutions per site, and node support was estimated by 10,000 ultrafast bootstrap replicates.

Maximum-likelihood phylogenies based on COI and 28S rRNA sequences placed Onchocercidae spp. Belitung I’ and ‘Belitung II’ in positions consistent with those inferred from the whole mitochondrial genome analyses (Figs 3 and 4). The COI sequences of *Dirofilaria* sp. ‘Sabah’ also fell within the clade formed by the genus *Dirofilaria*, and grouped monophyletically with *Dirofilaria* sp. ‘Thailand II’ and *D. asiatica* (Fig 2). Because 28S rRNA sequences for *D. asiatica* and *Dirofilaria* sp. ‘Thailand II’ were not available, their relationships could not be evaluated using nuclear genetic markers.

### Presence and absence of *Wolbachia* endosymbiont

DNA sequencing libraries from Onchocercidae sp. ‘Belitung I’, Onchocercidae sp. ‘Belitung II’ and *Dirofilaria* sp. ‘Sabah’ were screened for putative *Wolbachia-*derived sequences using the Chan Zuckerberg ID metagenomic pipeline [39]. *Wolbachia* DNA was detected in Onchocercidae sp. ‘Belitung I’ and *Dirofilaria* sp. ‘Sabah’, but not in Onchocercidae sp. ‘Belitung II’ (S3 Fig).

**Fig 5:**
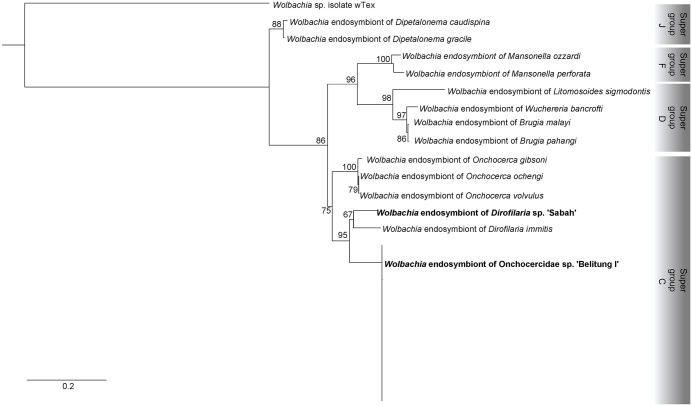
*Wolbachia* MLST genes (coxA, fbpA, and gatB) maximum likelihood phylogenetic tree. The scale bar represents 0.2 substitutions per site, and node support was estimated by 10,000 ultrafast bootstrap replicates [29].

Phylogenetic analysis of the *Wolbachia* MLST genes coxA, fbpA, and gatB placed both detected endosymbionts in supergroup C (Fig 5). The *Wolbachia* associated with Onchocercidae sp. ‘Belitung I’ formed a sister group to *Wolbachia* from *Dirofilaria* spp., whereas the *Wolbachia* associated with *Dirofilaria* sp. ‘Sabah’ was most closely related to that of *Dirofilaria immitis* (NZ_CP046578).

## Discussion

This study provides morphometric and molecular characterizations of Mf detected in potential reservoir hosts of *B. malayi* in Indonesia and Malaysia. Complete mitochondrial genomes, nuclear barcode regions, and *Wolbachia* marker genes were assembled and used in phylogenetic analyses that clarified the taxonomic placement of these previously uncharacterized filarial species within the family Onchocercidae and the genus *Dirofilaria*. These new molecular data highlight the extent of undescribed genetic and species diversity within Onchocercidae and expand public sequence databases that support molecular diagnostics.

The Onchocercidae sp. ‘Belitung I’ Mf were detected by microscopy in approximately 20% of the macaques. With an average length of around 500 µm, they are longer than any Mf known from humans and most commonly reported Mf from animals. To our knowledge, no molecularly described Mf match this size range. However, within the genus *Dirofilaria*, one species, *D. magnilarvatum*, was named to reflect the unusually large size of its unsheathed Mf (580.9 ± 10 µm) [46,47]. This species, which was described from *Macaca irus* (now *M. fascicularis*) in Malaysia, shows no periodicity and tends to accumulate in arterioles in the skin and tail. Because our Mf were found in the same host species, and natural or technical size variation is common, it is possible that ‘Belitung I’ corresponds to *D. magnilarvatum*. However, the absence of molecular data for *D. magilavatum* prevents definitive identification. Reported vectors for this species include *Mansonia bonneae, M. annulata* [48], *M. longipalpis,* and *M. uniformis* [49], which are also known vectors of *B. malayi*, a parasite also detected in these macaques [12]. A previous study in the same area reported Mf of a *Dirofilaria sp.* in macaques that may correspond to the species identified in our samples, although no morphological and molecular description were provided [50]. Taken together, these findings suggest that large Onchocercidae sp. ‘Belitung I’ Mf are relatively common in macaques on Belitung Island. Because parasites circulating in non-human primates may have zoonotic potential, further investigation of this lineage is warranted.

*Dirofilaria* sp. ‘Sabah’ morphologically resembled *D. asiatica* Mf. The Mf of both species had very similar lengths (299.1 µm vs. 300.3 µm), and the number of nuclei in the head space was also comparable (1–3 vs. 2–3) (S4 Table) [10]. However, because different staining methods were used, direct comparison should be interpreted with caution. Based on the COI region, *Dirofilaria* sp. ‘Sabah’ showed 97.4% identity to *D. asiatica* (2.6% divergence), within the intraspecific range proposed by Ferri et al. (2009) [51], although Ferri et al. used K2P distances rather than BLAST pairwise identity. However, mitochondrial genome phylogenetic analysis placed *Dirofilaria* sp. ‘Sabah’ in a distinct cluster, highlighting that COI genetic distance alone is insufficient for species delimitation. For example, *O. volvulus* and *O. ochengi* are readily distinguished by morphology and host specificity despite a mean interspecific divergence of only 1.9% [51]. The close relationship of the ‘Belitung I’ and particularly the ‘Sabah’ Mf to *D. asiatica* and *Dirofilaria* sp. ‘Thailand II’ suggests substantial diversity within the genus *Dirofilaria* in Asia [10,11,52]. Whether *Dirofilaria* sp. ‘Sabah’ represents a cryptic species, or a novel species can only be determined through the collection of adult worms and additional molecular data. Given its close phylogenetic proximity to *D. asiatica*, it is plausible that *Dirofilaria* sp. ‘Sabah’ shares similar epidemiological and ecological characteristics, including a zoonotic potential.

The Mf of Onchocercidae sp. ‘Belitung II’ are similar in size to *Mansonella* species [53]. Although whole mitochondrial genomes provide more robust phylogenetic information, their utility is limited because complete mitochondrial sequences are unavailable for many Filarioidea species. In our analyses, ‘Belitung II’ clustered as a sister group to *Mansonella*, a genus commonly found in humans and primates in Africa and South America [54,55]. The only *Mansonella* species described from Asia is *M. dunni*, reported from the common tree shrew (*Tupaia glis*) [56]. Because molecular data for non-human primate *Mansonella* species are lacking, other filarial species previously described in macaques, such as *Edesonfilaria malayensis* or *Macacanema formosana*, cannot be excluded (S4 Table). For example, the Mf of *M. formosana* found in Taiwanese macaques (*Macaca cyclopis*) measured 152 µm [57], closely resembling the length of ‘Belitung II’ (150 µm). Of particular interest is the low prevalence but extremely high Mf density (17,150 Mf/mL) of ‘Belitung II’, which may indicate highly efficient transmission by a specific vector species or immunosuppressive conditions in the affected macaque.

The *Wolbachia* profiles of the newly identified filarial species further support their inferred taxonomic relationships. *Wolbachia* from Onchocercidae sp. ‘Belitung I’ and *Dirofilaria* sp. ‘Sabah’ clustered within supergroup C and closely matched endosymbionts of *Onchocerca* and *Dirofilaria* species, findings that are broadly consistent with mitochondrial and nuclear phylogenies. In contrast, the absence of detectable *Wolbachia* in Onchocercidae sp. ‘Belitung II’ aligns with its placement near the *Wolbachia*-free species *Chandlerella quiscali* [58], although low infection levels may account for the negative results, as has been reported for other *Mansonella* species [59]. These findings require further experimental confirmation, such as immunohistochemistry and long-read sequencing, because the MLST marker gene analysis may have been confounded by sequences originating from nuclear *Wolbachia* transfers (nuwts). In addition, species that no longer harbor active *Wolbachia* infections may retain fragments of the *Wolbachia* genome that were incorporated into the nuclear genome during historical associations with the endosymbiont [60].

This study used mitochondrial, 28S rRNA, and *Wolbachia* MLST markers for molecular characterization, an approach that has inherent limitations. Although these markers are useful for species identification and phylogenetic analysis, they represent only a small fraction of the genome. Long-read sequencing or whole nuclear genome analysis could provide higher resolution and a more comprehensive understanding of genetic diversity and evolutionary relationships within this group. Although whole genome amplified DNA may be suitable for mitochondrial genome assembly or reference-based variant analysis when an appropriate reference genome is available, de novo nuclear genome assembly remains challenging. Chimeric reads generated through the fusion of noncontiguous DNA fragments during amplification can introduce scaffolding errors, resulting in misassemblies, fragmented contigs, and inaccurate genome reconstruction. These artifacts, together with uneven read coverage and amplification bias, further complicate reconstruction of the original nuclear genome. For these reasons, adult male specimens are often preferred for genome assembly because they can provide sufficient DNA for sequencing without amplification and do not contain embryonic DNA, which may interfere with assemblers that are typically optimized for diploid genomes.

For all three species described in this study, comprehensive information on critical aspects such as pathogenicity, vector competence, host preference, and geographical distribution is lacking. This absence of data represents a major gap in our understanding of filarial biocoenosis and underscores the need for further investigation.

## Conclusion

Analysis of individual Mf provides an effective approach for linking morphological features with genome data. Our findings highlight the limited knowledge of filarial species diversity in non-human primates and companion animals, and the need for more comprehensive genomic approaches to clarify their taxonomy. Although mitochondrial and 28S rRNA data provide useful information for species identification and phylogenetic analysis, future studies should include additional specimens and incorporate whole nuclear genome analyses to increase marker density and improve taxonomic resolution. The potential zoonotic relevance of these filarial species also underscores the need for further investigation, particularly to improve the diagnosis of *B. malayi*, which may be misidentified as one of the other species in endemic regions.

## Supporting information

S1 Table Metadata, microscopy, and qPCR results. (XLSX)

S2 Table NCBI accession numbers for sequences generated in this study. (XLSX)

S3 Table NCBI accession numbers for comparator sequences. (XLSX)

S4 Table Morphometric comparison of microfilaria across different filarial species. (XLSX)

S1 Fig Read coverage across the mitochondrial assemblies. (PDF)

S2 Fig Mitochondrial genome map showing gene contents and gene order. (PDF)

S3 Fig Relative abundance of *Wolbachia* sequences in DNA sequencing libraries from individual microfilariae. (PDF)

S1 Dataset Multiple sequence alignments and corresponding Newick tree files for all phylogenetic analyses. (ZIP)
