# Supplementary material for: Diverse Onchocercidae from Malaysian cats and Indonesian macaques: Morphological and molecular analysis of individual microfilariae using mitochondrial genomes, 28S rRNA, and Wolbachia endosymbiont sequences
Source: PLoS Negl Trop Dis. 2026 Jul 23;20(7):e0014015. doi: 10.1371/journal.pntd.0014015 (PMC13421770; doi:10.1371/journal.pntd.0014015)
Supplement: S2 Fig — (PDF) [file pntd.0014015.s006.pdf]

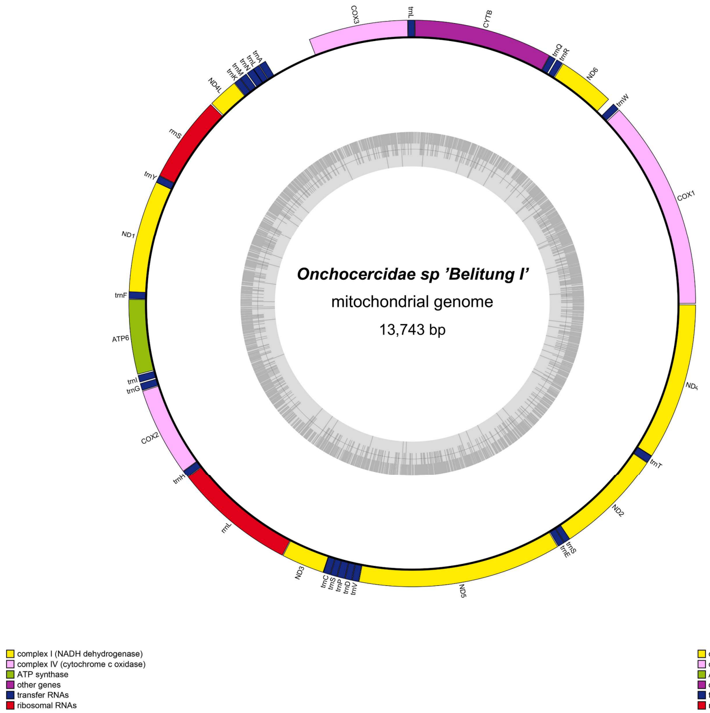
Onchocercidae sp 'Belitung I'
mitochondrial genome
13,743 bp
complex I (NADH dehydrogenase)
complex IV (cytochrome c oxidase)
ATP synthase
other genes
transfer RNAs
ribosomal RNAs

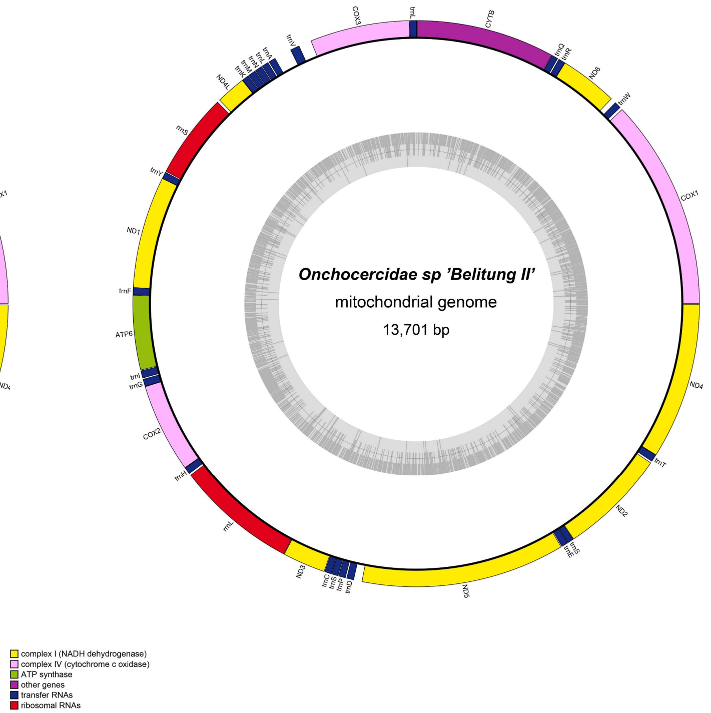
Onchocercidae sp 'Belitung II'
mitochondrial genome
13,701 bp
complex I (NADH dehydrogenase)
complex IV (cytochrome c oxidase)
ATP synthase
other genes
transfer RNAs
ribosomal RNAs

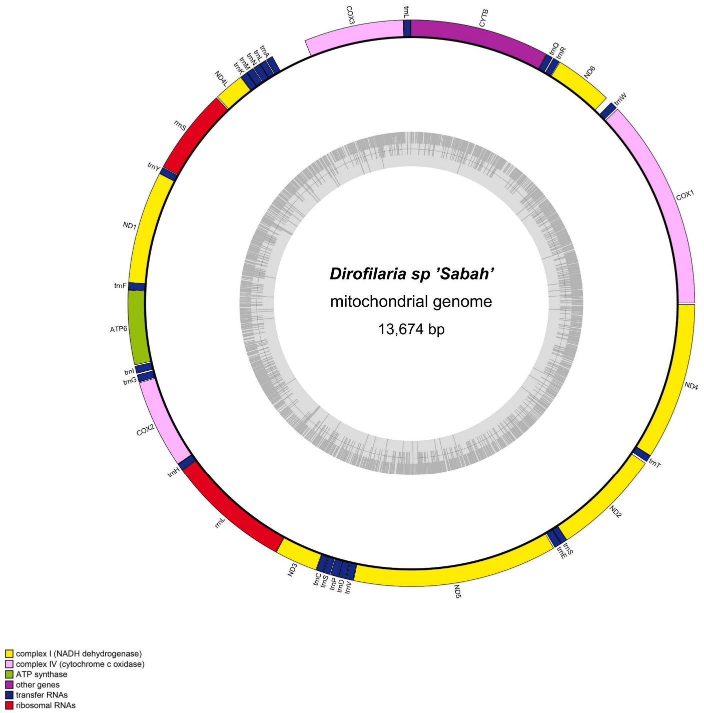
Dirofilaria sp 'Sabah'
mitochondrial genome
13,674 bp
complex I (NADH dehydrogenase)
complex IV (cytochrome c oxidase)
ATP synthase
other genes
transfer RNAs
ribosomal RNAs
