# Supplementary material for: Diverse Onchocercidae from Malaysian cats and Indonesian macaques: Morphological and molecular analysis of individual microfilariae using mitochondrial genomes, 28S rRNA, and Wolbachia endosymbiont sequences
Source: PLoS Negl Trop Dis. 2026 Jul 23;20(7):e0014015. doi: 10.1371/journal.pntd.0014015 (PMC13421770; doi:10.1371/journal.pntd.0014015)
Supplement: S3 Fig — (PDF) [file pntd.0014015.s007.pdf]

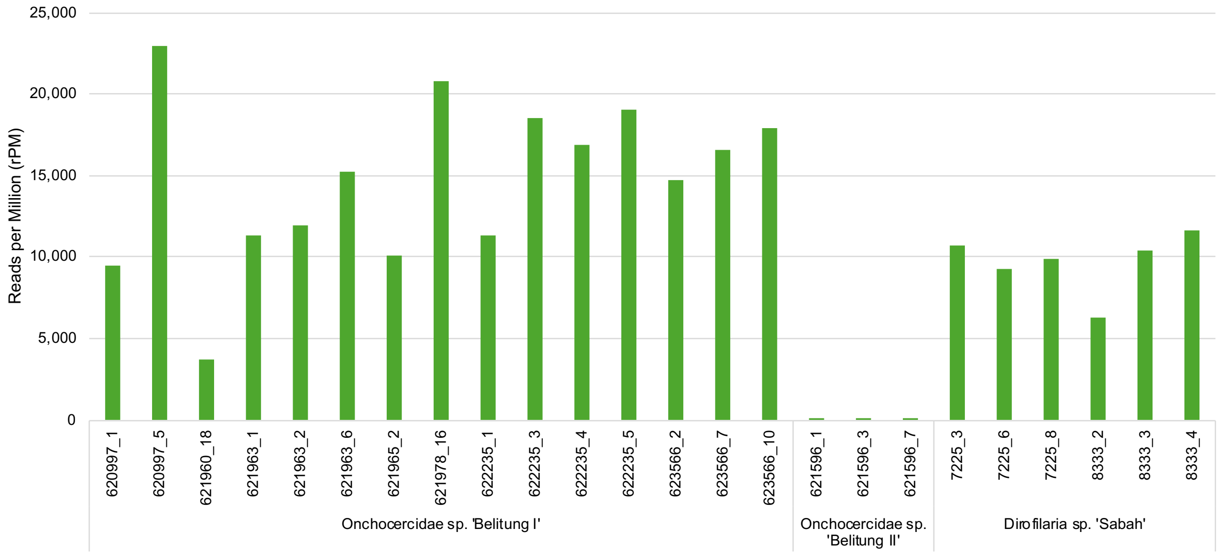


**S3 Fig.** Relative abundance of *Wolbachia* sequences in DNA sequencing libraries from individual microfilariae. The Chan Zuckerberg ID metagenomic pipeline was used for taxonomic identification and quantification.
